# Optimization of chaotic light output in semiconductor laser systems based on multi-objective optimization algorithm

**DOI:** 10.1371/journal.pone.0301630

**Published:** 2024-04-11

**Authors:** Jian Kong, Jinsong Li, Peng Li

**Affiliations:** 1 School of Physics and Optoelectronics Engineering, Anhui University, Hefei, China; 2 School of Information and Electronic Engineering, Lu’an Vocational and Technical College, Lu’an, China; Federal University of Technology - Parana, BRAZIL

## Abstract

Aiming at the weak performance of chaotic light output in semiconductor laser systems, the study designed a power control algorithm for semiconductor laser drive systems based on linear self-disturbance rejection control. Then the optimization parameters and scope were determined, and multi-objective optimization and direction preference algorithms were introduced. A chaotic optical performance optimization model based on improved multi-objective genetic algorithm was constructed using adaptive functions as evaluation indicators. These results confirmed that the larger the bandwidth of the controller, the faster the response speed of the resonant converter, but the stability was poor. When the input voltage underwent a sudden change, the current ripple coefficient of the PID algorithm was 0.55%. The linear active disturbance rejection control algorithm could ensure that the voltage and current maintained at the set values, and the output current of the algorithm was more stable when the load underwent sudden changes. The directional preference algorithm could further provide more valuable solutions on the basis of adaptive genetic algorithms. When the peak value of the autocorrelation function was equal to 0.2, the delay characteristics of chaotic light were effectively suppressed, having strong signal bandwidth and complexity. In summary, the constructed model has good application effects in optimizing chaotic optical performance and has certain positive significance for communication security.

## 1. Introduction

With the development of technology, optical communication develops rapidly, and information security receives more attention. Chaotic light is an optical technology that utilizes the nonlinear characteristics of light by adding chaotic signals to the light, making the transmitted optical signals highly uncertain and complex. The anti-eavesdropping performance of optical signals can be improved and the security of key transmission and has broad application prospects in fields such as optical communication, quantum communication, and information security [1]. The improvement of chaotic optical performance not only enhances the security of information transmission, but also helps to improve the performance of optical communication systems [2]. Renewable energy has received increasing attention with the increasing demand for energy worldwide [3]. Semiconductor Laser System (SLS) uses semiconductor materials as working materials and can generate high-energy lasers using solar energy or other renewable energy sources, making important contributions to the development of renewable energy [4]. However, at present, the efficiency of semiconductor lasers in output chaotic light is relatively low, and chaotic light signals are usually generated through nonlinear optical effects, which requires higher requirements for lasers. In addition, there are some problems with the spectral width and dynamic range of chaotic light output by semiconductor lasers, which directly affect signal transmission and data capacity. At present, the chaotic light output by SLS usually has problems of narrow spectral width and limited dynamic range, which limit the performance in high-speed data transmission and other applications [5]. In this context, a power control algorithm for semiconductor laser drive systems was studied, and a chaotic optical performance optimization model based on Multi-objective Genetic Algorithm (MOGA) was constructed. The main structure of the study consists of four parts. Firstly, an analysis is conducted on the current research status. Secondly, a power control algorithm for the semiconductor laser driving system is designed, and a chaotic optical performance optimization model based on the improved MOGA is constructed. The third part is to analyze the application effect of the proposed model. Finally, there is a summary of the entire study.

The innovation of this study mainly involves two points. Firstly, a power control algorithm for semiconductor laser driving system is proposed to address the control problem of the driving system. Secondly, MOGA and the directional preference algorithm are used to optimize the performance of chaotic light.

The objective of this study is to propose a power control algorithm for semiconductor laser drive systems and a chaotic optical performance optimization model based on MOGA. So the output efficiency and performance of chaotic light can be further improved, and the needs of practical applications can be better met.

The main contribution of this study is to address the control problems of high-frequency semiconductor laser drive systems and the relatively low efficiency of the output chaotic light, thereby improving the stability of chaotic light and optimizing the performance. This study is expected to further enhance the security of information transmission, increase the confidentiality and anti-interference ability of communication, and effectively prevent information from being eavesdropped, damaged, or tampered applied in fields such as optical communication, quantum communication, and information security. In addition, this study is expected to provide new ideas and methods for engineering technology, improve the power control efficiency and chaos optical performance optimization of semiconductor laser drive systems, and promote the benefits of related technologies in practical applications. This study can also provide new theoretical foundations and methods for relevant academic research to a certain extent, which helps to promote the development and deepening of the discipline.

## 2. Related works

Multi-objective Optimization (MOP) refers to the process of simultaneously optimizing multiple objectives in a given region as much as possible, without a unique global optimal solution. The solution of MOP is usually a set of optimal solutions composed of numerous Pareto optimal solutions. C. Lu et al. proposed a Pareto-based collaborative MOP for energy-saving scheduling of distributed arranged flow shop problems with limited buffers and compared this with other MOP methods in practice. These results confirmed that the proposed algorithm had good convergence and certain effectiveness and could achieve excellent results on all problems related to comprehensive metrics [6]. C. Mokhtara et al. proposed an optimization method for a hybrid renewable energy system for rural residential electrification using particle swarm optimization algorithm. Meanwhile, ε-constraint method was used to solve MOP problems, aiming at minimizing energy costs while maximizing system reliability and renewable parts. The results indicated that the proposed MOP method had good optimization performance [7]. The research of Y. Tian et al. had shown good performance in solving various optimization problems with MOP, but the performance might deteriorate sharply when dealing with problems involving a large number of decision variables. A comprehensive review was conducted on the latest MOP, discussing their respective advantages and disadvantages and pointing out the challenges faced by MOP and future research directions [8]. J. Wang et al. designed a multi-layer encoding strategy and constraint scheduling method to address the allocation of multiple unmanned aerial vehicles in complex tasks, addressing the key logical and physical constraints. An improved multi-objective quantum behavior method was proposed to effectively solve MOP. These results confirmed that the proposed model and algorithm achieved feasibility and effectiveness [9]. J Martinez Rico et al. proposed an energy arbitrage strategy and fitted a multi-objective method to reduce the value loss of batteries by considering the role of batteries in compensating for the randomness and intermittency of renewable energy. The results indicated that the use of multi-objective cost functions could significantly increase battery life without affecting profitability [10]. D. Dinh Cong et al. proposed a search algorithm-based MOP to identify the location and degree of multiple damages in functionally graded material structures to solve the limited application of MOP in damage detection of composite material structures. These results confirmed that the proposed algorithm had good performance in damage detection and prediction, which achieved feasibility and effectiveness [11]. P. Jangir et al. proposed a multi-objective ocean predator algorithm to solve the multiple conflicting objectives, and this algorithm was tested in various multi-objective cases. These results confirmed that the proposed algorithm had certain effectiveness [12].

Chaotic optical signals are highly complex and random optical signals that can achieve secure optical communication and data transmission. A. F. Tazay et al. conducted a detailed feasibility analysis of hybrid renewable energy systems to provide sufficient energy for the buildings of autonomous colleges. The feasibility analysis proposed the technical and economic forms of hybrid renewable energy systems, including photovoltaics. The results indicated that photovoltaic systems had a significant impact on the net present value cost and energy cost [13]. M. Zhang et al. stated that chaotic optical signals had extensive applications in fields such as secure communication, random number generation, and optical sensing. The latest developments were outlined in high-performance chaos generation and the unique measurement applications, and several innovative methods that supported chaos optimization were discussed. They also looked forward to the future research and development directions of chaotic laser and chaotic measurement technology [14]. Z. Gao et al. proposed and experimentally demonstrated a new high-speed physical security key distribution scheme based on chaotic optical signal processing and dedicated hardware modules to address the technical challenges of fiber optical communication. A promising strategy was provided for high-speed key distribution based on chaotic optical signal processing and classical fiber channels [15]. V. Annovazzi Lodi et al. demonstrated two chaotic systems, each consisting of two coupled semiconductor lasers, which were synchronized using direct optical feedback. The robustness of the proposed synchronization scheme to source parameter mismatch and startup condition differences was tested through numerical simulation. The application of chaos masking and chaos shift keying in secure data transmission was proposed [16]. M. G. L. Roes et al. proposed a dual output resonant converter control strategy based on disturbance observer to solve the high cost of feedback sensor isolation, and the current control of two LED loads was achieved through estimation. The entire implementation aimed at a low cost solution [17]. C. Buccella et al. established a nonlinear model of LLC resonant converter using the extended description function. A controller based on a nonlinear observer was designed and implemented. The results indicated that the proposed observer-based controller achieved good stability [18].

An inverter can convert direct current energy into fixed frequency and voltage or frequency and voltage regulated alternating current. A resonant converter is a new type of inverter. K. Suresh et al. proposed an improved multilevel inverter that used fewer components for boost operation to reduce the total harmonic distortion, addressing the issue that existing multilevel inverters could not perform boost operation [19]. A. D. Falehi et al. proposed a fractional order super twisted sliding mode control for a hybrid energy storage system of lithium batteries and supercapacitors to deal with the controller design of dynamic voltage restorers. So the complex correlation, unknown nonlinearity, and parameter uncertainty between system components were compensated [20]. Meanwhile, a creative structure for was proposed inverters based on a series connected power electronic compensator type dynamic voltage restorer to improve the fault crossing ability and power quality of doubly fed induction generators [21]. A. D. Falehi proposed a novel hybrid multilevel inverter coupled with photovoltaic power sources as an AC voltage synthesizer for dynamic voltage restorers to accurately compensate for any voltage disturbances [22]. Zhao et al. proposed a hybrid inverter combining switch capacitor units and flying capacitor structures to reduce the components and achieve self-balancing of capacitors, which reduced current stress, simplified control, and boosted capabilities. The experimental results verified the feasibility of the topology structure [23]. M. Lu et al. studied a class of second-order circuits composed of harmonic oscillators and nonlinear state-dependent damping. Analysis methods based on average and perturbation theory were outlined to derive several performance indicators related to the harmonic and dynamic characteristics of these oscillators within a unified framework. It was beneficial for the application of inverters in power grid formation [24].

In summary, although many experts and scholars have conducted extensive research on chaotic optical signals, the chaotic optical performance output by SLS is still weak. Therefore, a power control algorithm for semiconductor laser driving system was proposed, and MOP was used to optimize the chaotic optical performance of SLS output to improve the security of information transmission.

## 3. Chaotic optical performance optimization based on multi-objective GA

The optimization of the chaotic optical performance of SLS output is crucial to improve the security of information transmission. To improve stability of chaotic light and optimize the performance of chaotic light, this study innovatively proposes a power control algorithm for semiconductor laser drive system to control the semiconductor laser. Multi-objective genetic optimization algorithm and directional preference algorithm are used to optimize the chaotic optical performance.

### 3.1. Power control algorithm for semiconductor laser drive system

**Fig 1:**
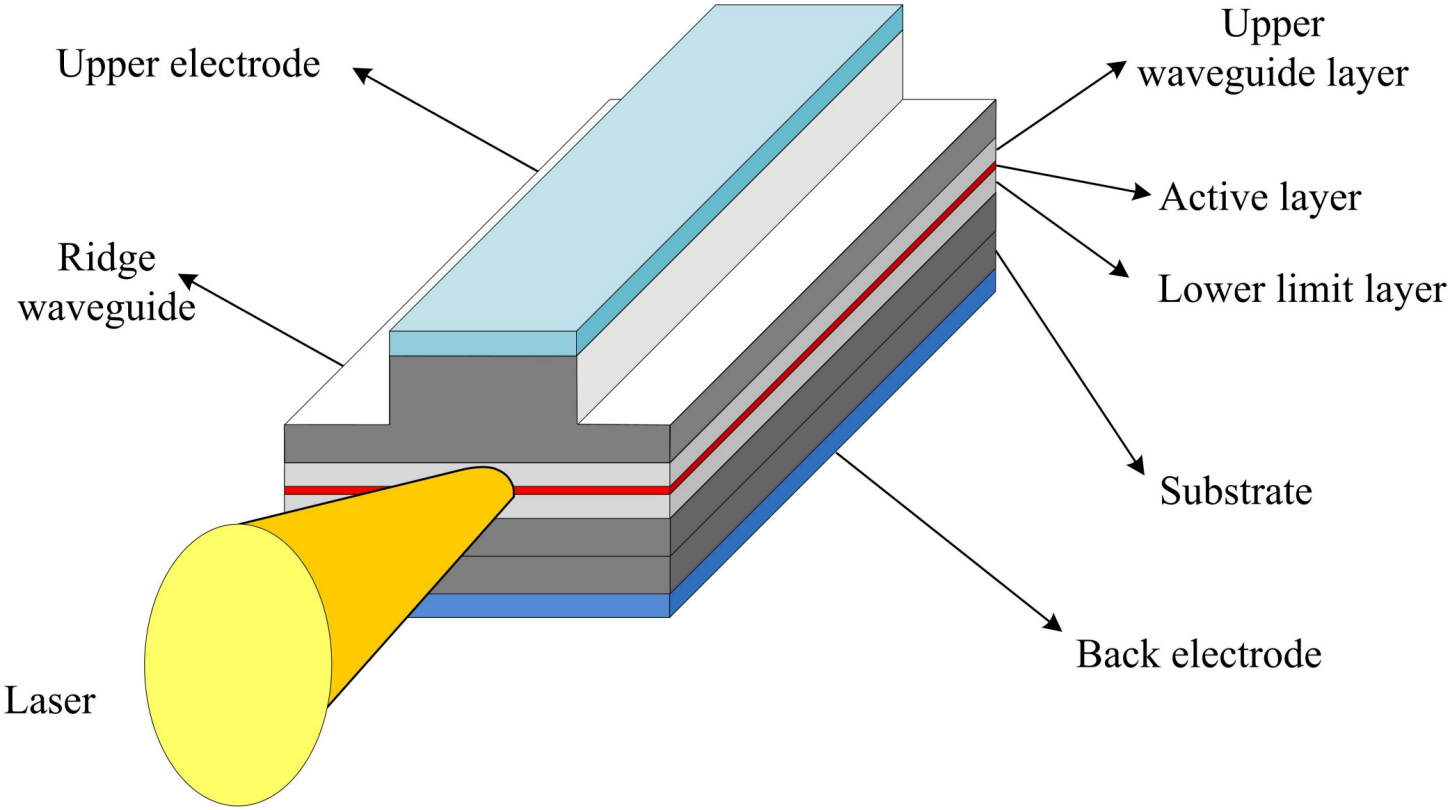
Schematic diagram of the structure of a semiconductor laser.

Laser is a highly concentrated, coherent, monochromatic, and directional light generated through stimulated radiation. Laser is stimulated in an activated medium, amplified by an optical resonant cavity. Ultimately, a strong and highly consistent beam of light is produced. A semiconductor laser, also known as a laser diode, is a device that generates a laser beam. The working material is usually a semiconductor material, and the necessary conditions for generating laser are a gain medium, an excitation source, and a stable optical resonant cavity. Semiconductor lasers have the advantages of simple structure, long lifespan, easy modulation, and low cost. There are mainly three excitation methods: optical pumping, electric injection, and high-energy electron beam excitation. There are various structures of semiconductor lasers, which are determined by the epitaxial structure on the substrate chip. Fig 1 shows the structure of a semiconductor laser.

**Fig 2:**
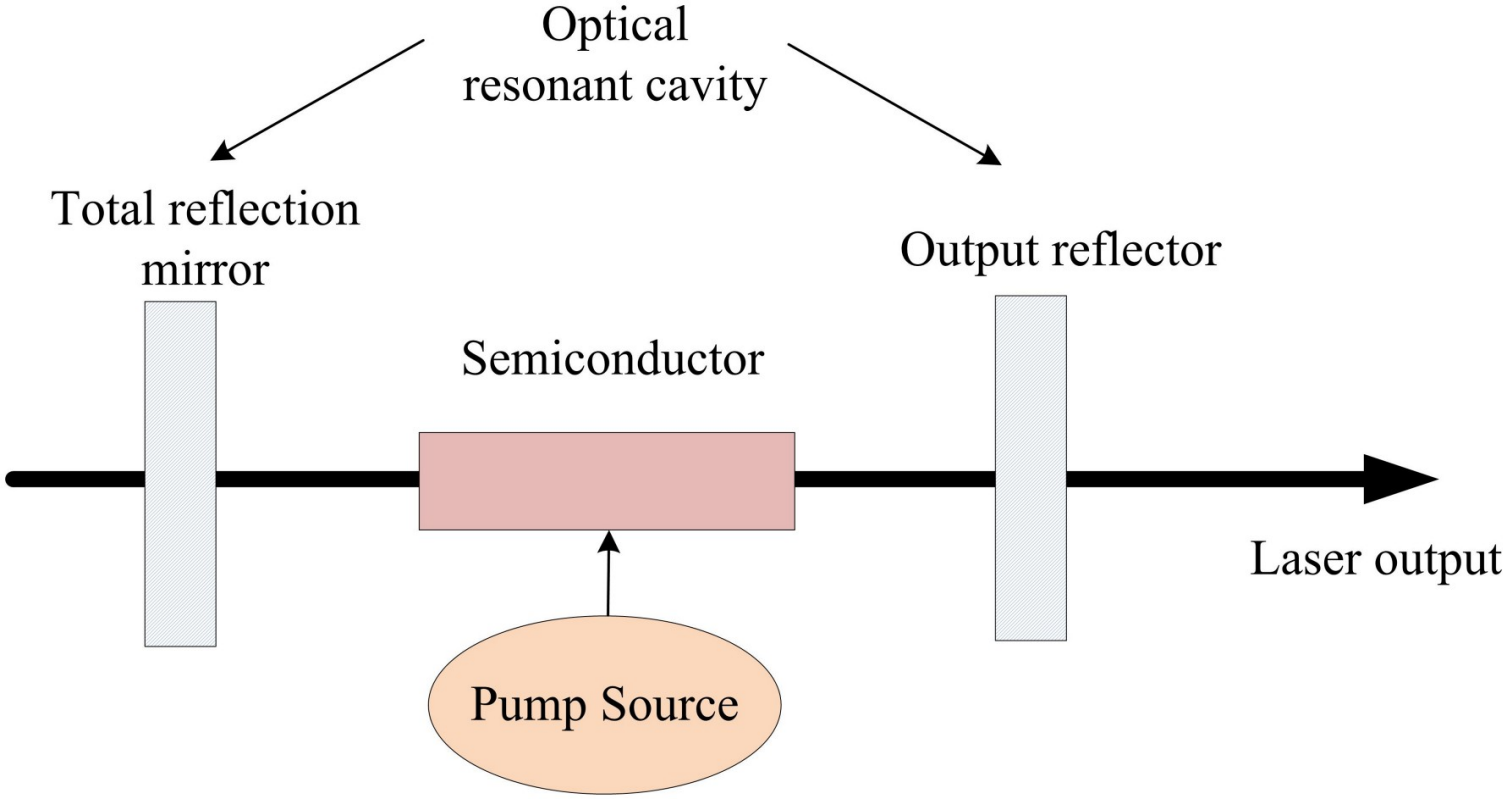
Schematic diagram of chaotic light output from semiconductor laser system.

With the advancement of semiconductor laser technology, high-power semiconductor lasers have emerged. The driving power supply of high-power semiconductor lasers requires constant current source characteristics and high requirements for current ripple. The power control of the driving system can affect the current and power output of semiconductor lasers, thereby affecting the performance and characteristics of chaotic light. By optimizing the power control of the semiconductor laser driven system, the stability, spectrum width, and dynamic range of chaotic light can be improved, and noise and nonlinear effects can be reduced [25]. Therefore, the power control of semiconductor laser driving system is very important for optimizing the chaotic optical properties. The schematic diagram of the SLS outputting chaotic light is shown in Fig 2.

**Fig 3:**
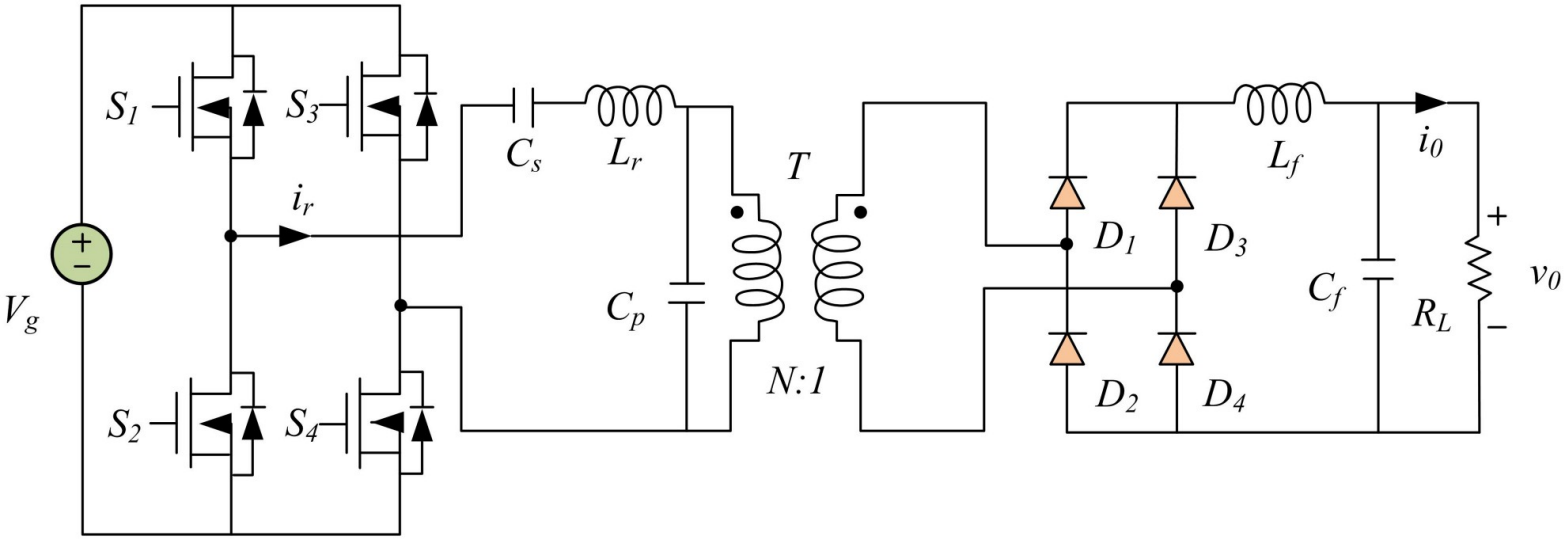
Topological structure of LCC resonant converter.

In the design of the main power supply for semiconductor laser drive systems, the disturbances in input voltage or external signals may lead to unstable output current. Therefore, an LCC resonant converter is used as the topology of the main power circuit. Resonant technology can make the voltage and current of devices vary in a sinusoidal manner. Resonant converters are mainly composed of switch networks, resonant slots, low-pass filters, and rectifier networks. LCC resonant converter is a series parallel resonant converter. LCC resonant converters not only inherit the advantages of both, but also overcome their shortcomings compared to traditional series and parallel resonant converters. LCC resonant converter has good constant current source characteristics, a wider range of voltage and current regulation, and strong resistance to output short circuits, which is suitable for high-power semiconductor laser-driven power supply topology. The operating mode of LCC resonant converter can be controlled by adjusting the switching frequency and resonant frequency. The resonant converter operates in intermittent current mode and can be applied to small and medium power outputs when the switching frequency is less than half of the resonant frequency. The resonant converter operates in continuous current mode, the LCC resonant cavity is capacitive, and the resonant current is continuous when the switching frequency is between half of the resonant frequency and the resonant frequency. The resonant converter operates in continuous current mode, the resonant current lags behind the voltage, and the LCC resonant cavity is in an inductive state when the switching frequency is greater than the resonant frequency. The operating frequency is equivalent to the parallel resonant frequency when working in an overloaded state. The operating frequency is equivalent to the series resonant frequency when working in a light load state. Fig 3 shows the topology of the LCC resonant converter. In Fig 3, V_g_ represents the input voltage, S means the switching transistor, i_r_ is the resonant current, C_s_ refers to the resonant series capacitor, L_r_ represents the series resonant inductor, C_p_ means the parallel resonant capacitor, T is a transformer, D refers to the rectifier diode, L_f_ represents the filter, C_f_ means the filter capacitor, i_0_ is the output current, R_L_ refers to the load, and v_0_ represents the output voltage.

The mathematical model of LCC resonant converter has a strong nonlinearity, which is not conducive to analysis. Therefore, the nonlinear model should be linearized first. According to the fundamental approximation method and the theory of linear differential equations, if the excitation signal changes in a sinusoidal state, the special solution of the equation and the excitation signal have the same form. The various state variables in the resonant network are decomposed into the superposition of a time-varying sinusoidal signal and a cosine signal, as shown in formula (1).


$$\left\{\begin{array}{c} i_{r}\approx i_{rs}(t)\;\text{sin}\;\omega_{s}t+i_{rc}(t)\;\text{cos}\;\omega_{s}t\\ v_{cs}\approx v_{CsS}(t)\;\text{sin}\;\omega_{s}t+v_{CsS}(t)\;\text{cos}\;\omega_{s}t\\ vC_{p}^{\prime }\approx vC_{p}^{\prime }s(t)\;\text{sin}\;\omega_{s}t+vC_{p}^{\prime }c(t)\;\text{cos}\;\omega_{s}t \end{array}\right.$$ (1)

In formula (1), *i*_*r*_, *v*_*Cs*_, and $vC_{p}^{\prime }$ represent the state parameters of the resonant converter, and $C_{p}^{\prime }$ represents the capacitance value of the parallel capacitor. *i*_*rs*_, *i*_*rc*_, *v*_*CsS*_, *v*_C*s*C_, $vC_{p}^{\prime }s$, and $vC_{p}^{\prime }c$ are constants. The remaining derivatives are 0 when the resonant network input of the LCC resonant converter is an AC sine voltage and the resonant network enters a stable state. For the three nonlinear terms contained in formula (1), an extended descriptive function approximation is used to transform the nonlinear term into an approximate linear term, and formula (2) is obtained by Fourier expansion.


$$\left\{\begin{array}{c} v_{AB}=M(d,v_{g})\;\text{sin}\;\omega_{s}t=\frac{4v_{g}}{\pi }\;\text{sin}\;(\frac{d}{2})\;\text{sin}\;\omega_{s}t\\ sign(vC_{p}^{\prime })i_{L_{f}}=\frac{4i_{L_{f}}}{\pi A_{p}}(vC_{p}^{\prime }s\;\text{sin}\;\omega_{s}t+vC_{p}^{\prime }c\;\text{cos}\;\omega_{s}t)\\ \left|vC_{p}^{\prime }\right|=M(vC_{p}^{\prime }s,vC_{p}^{\prime }c)=\frac{2}{\pi }Ap\\ Ap=\sqrt{v_{C_{p}^{\prime }s}^{2}+v_{C_{p}^{\prime }c}^{2}} \end{array}\right.$$ (2)

In formula (2), *M* represents the extended descriptive function. The traditional semiconductor laser driving system adopts PID control, which has the advantages of simple structure and simple operation [26]. In a continuous time domain, formula (3) is the general form of a PID controller.


$$u(t)=K_{p}e(t)+K_{I}e(t)\int e(t)dt+K_{D}\frac{de(t)}{dt}$$ (3)

In formula (3), *u*(*t*) represents the controller output. *K*_*p*_ represents the proportional coefficient. *e*(*t*) represents feedback error. *K*_*I*_ represents the integration coefficient. *K*_*D*_ represents the differential coefficient. The transfer function of LCC resonant converter has second-order double poles caused by beat frequency, leading to a 180° hysteresis phase shift in the system. If the influence cannot be effectively suppressed, the influence will affect the adjustment speed, leading to the instability of the system. The usual approach is to design a compensation network to counteract the influence of dual poles. However, the PID controller overly relies on the system model in parameter design, which is difficult to meet the requirements in complex systems. Moreover, PID controllers are prone to integral saturation, which affect the control performance and have poor anti-interference ability. Therefore, Linear Active Disturbance Rejection Control (LADRC) is proposed to suppress the instability of current caused by multi-factor disturbances. LADRC is a control algorithm that does not rely on the object model. The bandwidth of LCC resonant converters is usually designed in the tens to hundreds of kilohertz range. The high-frequency zeros and poles of the system are attenuated outside the bandwidth. Therefore, the system can be simplified as a second-order model. Formula (4) is the output of the main power supply LCC resonant converter system driven by a high-power semiconductor laser.


$$y\prime \prime =p(t,y,\dot{y},\omega )+b_{0}u$$ (4)

In formula (4), *y* represents the output. *p* represents a function that drives the state of the system. *t* represents the time. *ω* represents the disturbance. *b*_0_ represents the disturbance compensation. *u* represents the input. *p* is mainly affected by the various derivatives of the input and output of the main power supply system, as well as the unknown internal dynamic disturbance information. Assuming that *x*_1_ = *y*, $x_{2}=\dot{y}$, *x*_3_ = *p*, *x* = [*x*_1_, *x*_2_, *x*_3_]^*T*^, and the total disturbance *p* is differentiable, the system vector is expressed as formula (5).


$$\left\{\begin{array}{c} \dot{x}=Ax+Bu+E\dot{p}\\ y=Cx \end{array}\right.$$ (5)

In formula (5), $A=\left[\begin{array}{c} 0,1,0\\ 0,0,1\\ 0,0,0 \end{array}\right]$, *B* = [*b*_0_, 0, 0]^*T*^, *E* = [0, 0, 0]^*T*^, and *C* = [1, 0, 0]^*T*^. The total disturbance of the system can be estimated by a linear expansion observer, as shown in formula (6).


$$\left\{\begin{array}{c} \dot{z}=Az+Bu+L(y-\hat{y})\\ \hat{y}=Cz \end{array}\right.$$ (6)

In formula (6), *L* represents the gain matrix of the linear expansion observer. $\hat{y}$ represents the estimated value of the system output, *z* represents the state variable of the linear expansion observer. When the adoption number *L* is accurately adjusted, the error coefficient matrix of formula (4) will gradually stabilize under bounded conditions. The transfer function of LCC resonant converter can be equivalent to a second-order system. Therefore, a third-order linear expansion observer is designed to observe the state and total disturbance of LCC resonant converter in formula (7).


$$\left\{\begin{array}{c} {\dot{z}}_{1}=z_{2}+l_{1}(y-z_{1})\\ {\dot{z}}_{2}=z_{3}+l_{2}(y-z_{1})+b_{0}u\\ {\dot{z}}_{3}=l_{3}(y-z_{1}) \end{array}\right.$$ (7)

In formula (7), *l* represents the gain of the observer. Therefore, formula (8) is the characteristic polynomial of the extended observer.


$$\left\{\begin{array}{c} \left|sI-(A-LC)\right|=s^{3}+l_{1}s^{2}+l_{2}s+l_{3}\\ A-LC=\left[\begin{array}{c} -l_{1},1,0\\ -l_{2},0,1\\ -l_{3},0,0 \end{array}\right] \end{array}\right.$$ (8)

In formula (8), *A* − *LC* represents the system matrix corresponding to the extended observer. According to the bandwidth method, formula (9) is considered an ideal equation.


$$s^{3}+l_{1}s^{2}+l_{2}s+l_{3}={\left(s+\omega_{o}\right)}^{3}$$ (9)

In formula (9), *ω*_*o*_ represents the observer bandwidth. The parameter tuning can be transformed into the selection of the observer bandwidth by assigning all poles of the extended observer to the left half plane. The control rate in formula (10) is usually used to compensate for the unknown disturbances in the system.


$$u=\frac{u_{0}-z_{3}}{b_{0}}$$ (10)

In formula (10), *u*_0_ represents the output of the linear state error feedback rate. *z* represents the observer state. The system is reconstructed through disturbance compensation by introducing formula (9) into formula (10), which can be approximated as a pure integral series type. By designing a linear error feedback rate for control, the system can achieve satisfactory control performance. Formula (11) is the design of the controller.


$$\left\{\begin{array}{c} u_{0}=k_{p}(r-z_{1})-k_{d}z_{2}\\ k_{p}=\omega_{c}^{2},k_{d}=2\omega_{c} \end{array}\right.$$ (11)

**Fig 4:**
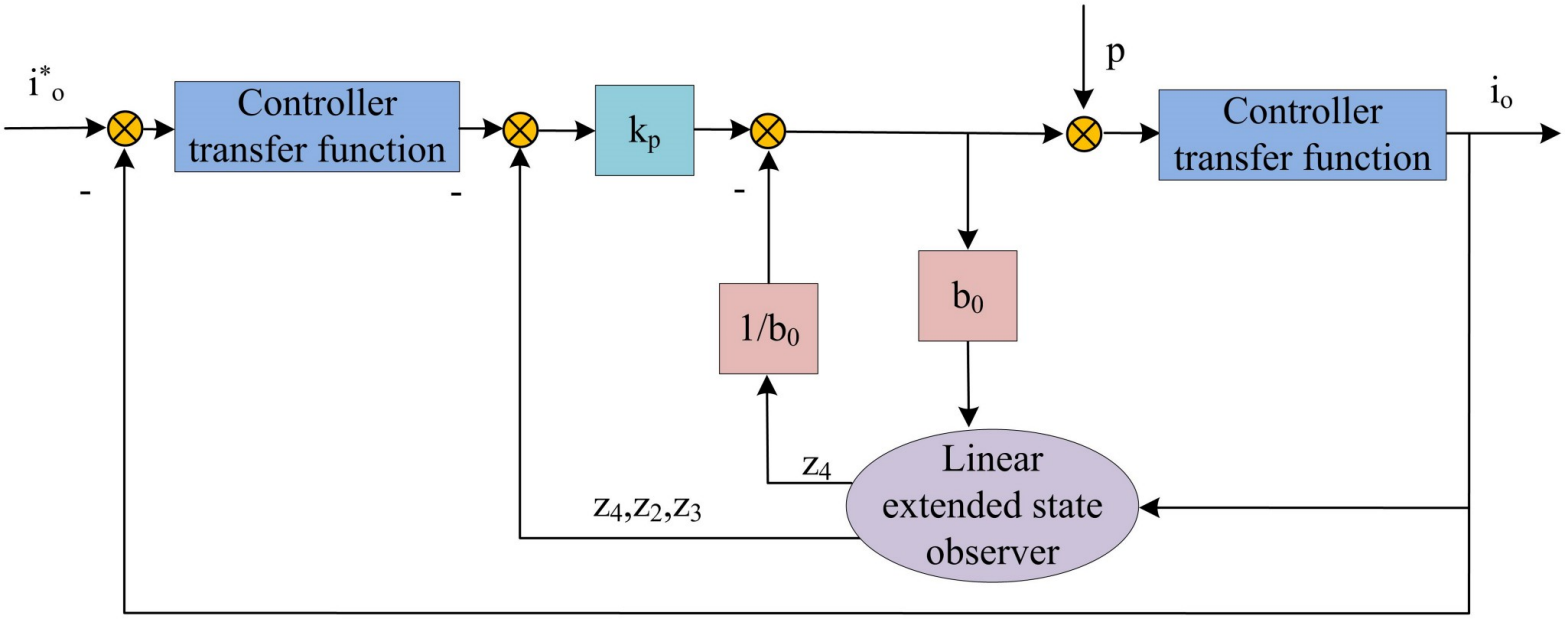
LADRC framework diagram.

In formula (11), *k*_*p*_ and *k*_*d*_ represent the gains of the controller. *r* represents a given value. *ω*_*c*_ represents the controller bandwidth, which determines the response speed of the controller. In summary, the design of LADRC does not rely on the accurate mathematical model of the LCC resonant converter, and system disturbances can be observed and tracked in real-time. Fig 4 shows the specific framework. In Fig 4, $i_{0}^{*}$ represents the input, *K*_*p*_ represents the scaling coefficient, *b*_0_ represents the estimated input gain, and *p* represents the external disturbance.

### 3.2. The construction of chaotic light optimization model based on multi-objective GA

The optical injection optical feedback SLS has a wide range of applications and multiple internal parameters, which is suitable for optimization using MOP algorithms. Therefore, this study focuses on optimizing the optical injection optical feedback SLS. The optimization parameters and scope should be determined first before optimizing the MOGA design for the performance of the semiconductor laser output chaotic light. The parameters for optimizing chaotic optical performance by optimizing the parameter range of SLS mainly include injection intensity, detuning frequency, feedback intensity, laser external cavity length, and linear broadening factor. Table 1 shows the SLS parameters and their optimization ranges.

**Table 1:** Semiconductor laser system parameters and parameter optimization table.

Parameter	Region
Feedback intensity/ns	[0,70]
Injected current	[1,2]
Detuning frequency/GHz	[–20,20]
Line-width enhancement factor	[1,5]
Laser external cavity length/m	[0.1,1]

**Fig 5:**
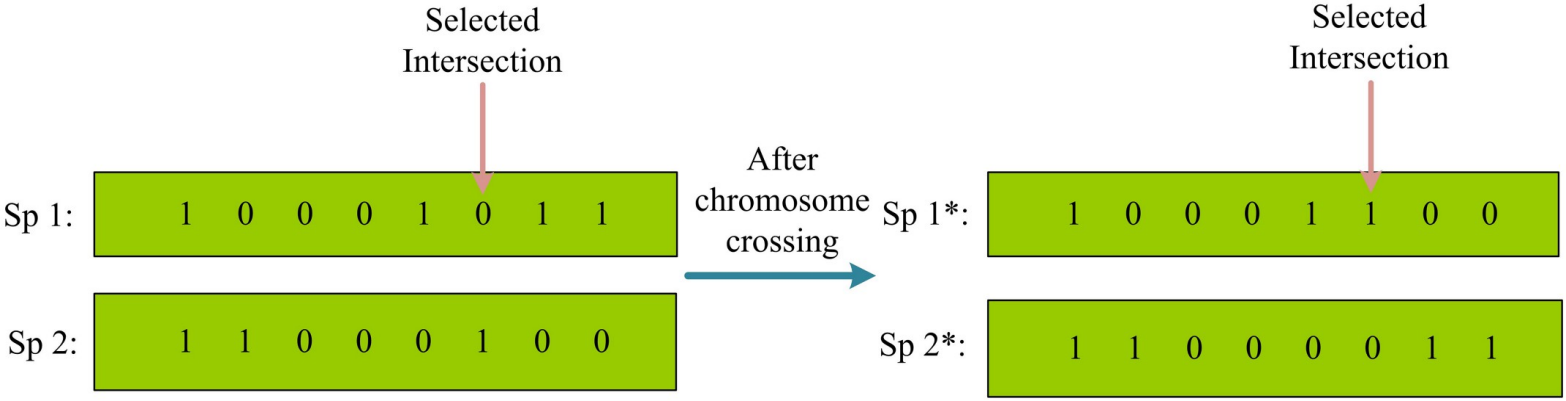
Schematic diagram of single-point crossing.

Optimization problems are often multi-attribute. Therefore, MOP appears. MOP compromises multiple conflicting targets to obtain a noninferior solution set. MOGA is currently one of the widely used MOP. MOGA is commonly used to solve multi-objective decision-making problems, which can simultaneously consider multiple objectives and find a set of optimal solutions. MOGA has good global optimization performance and strong adaptability, which can overcome the mutual constraints between various objective functions. Therefore, MOGA is used in this study to find the Pareto frontier of the optimal solution and model the system [27]. The basic steps of Genetic Algorithm (GA) include encoding, decoding, setting the initial population, determining fitness functions. GA has selection, crossover, and mutation operators. The crossover operation in GA is similar to gene recombination in nature and is the main method for generating new individuals, which is beneficial for improving the search ability and efficiency of the algorithm. This operation mainly involves selecting the superior genes from the parent genes for hybridization to ensure that the genes of the new individual are superior to those of the parents. Choosing different crossover methods will result in different genetic individuals [28]. The commonly used crossover operators include single-point crossover and two-point crossover. Fig 5 shows a single-point intersection.

MOP often has multiple optimal solutions, so multiple evaluation functions are often needed in GA to evaluate individuals and construct the optimal solution set. Given the decision vector *X* = (*x*_1_, *x*_2_, …, *x*_*n*_), when there are *n* optimization objectives, they usually conflict with each other. Formula (12) is the optimization objective.


$$f(X)=(f_{1}(X),f_{2}(X),\ldots ,f_{n}(X))$$ (12)

Different optimization problems often have different optimization objectives, which can cause complex operations. The orientation of all optimization objectives is usually uniformly processed to simplify operations. The minimized objective function can be transformed into the maximized one, or the maximized objective function can be transformed into the minimized one in formula (13).


$$\left\{\begin{array}{c} \text{min}\;f_{i}(X)=-\text{max}(-f_{i}(X))\\ \text{max}\;f_{i}(X)=-\text{min}(-f_{i}(X)) \end{array}\right.$$ (13)

The current methods for constructing Pareto solution sets for multi-objective problems include nondominated sorting, challenge arena, and Zhuang family methods. The Nondominated Sorting Genetic Algorithm-II (NSGA-II) selected in this study can preserve better solutions without being destroyed by genetic mutation operations. NSGA-II is an evolutionary algorithm based on GA, aimed at finding the Pareto optimal solution set of problems. Based on GA, the concepts of nondominated sorting and crowding distance are introduced to deal with MOP. The core idea of this algorithm is to sort the individuals in the population according to nondominance, making the higher ranked individuals more advantageous. At the same time, NSGA-II maintains population diversity by calculating the crowding distance of individuals to avoid focusing solely on a single-solution set. The operation of NSGA-II mainly includes initial population generation, selection, crossover, mutation, and nondominated sorting. NSGA-II is widely used in engineering design, path planning, combinatorial optimization, and other fields. Formula (14) transforms the performance calculation functions of the three aspects of chaotic light into evaluation functions with consistent optimization directions.


$$\left\{\begin{array}{c} f_{1}=\Delta f_{ed}\\ f_{2}=\frac{1}{P_{ac}+1}\\ f_{1}=\lambda_{\;\text{max}} \end{array}\right.$$ (14)

**Fig 6:**
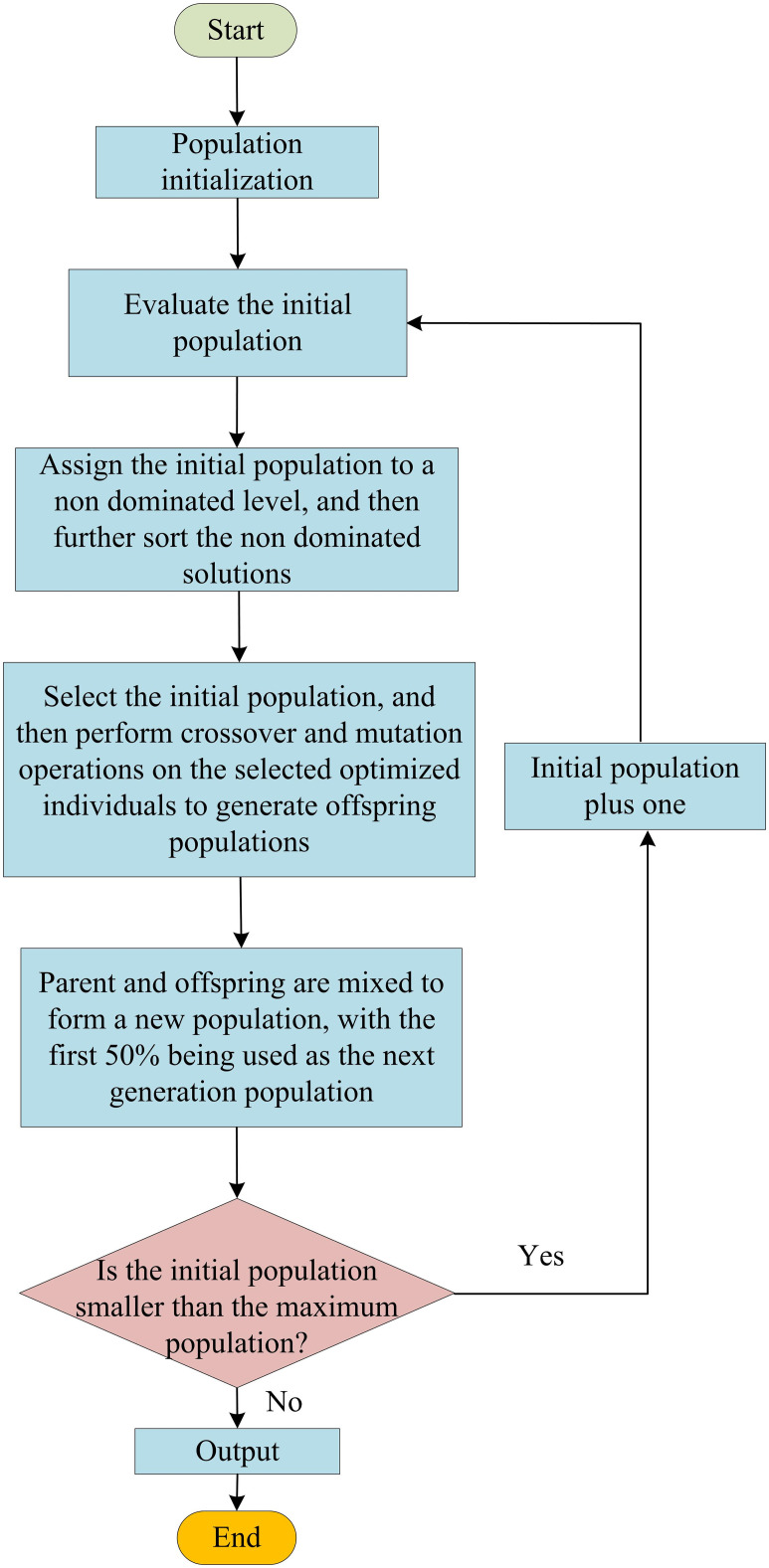
Flowchart of chaos light optimization model based on MOGA.

In formula (14), Δ*f*_*ed*_ represents the effective bandwidth. *P*_*ac*_ represents the peak of the autocorrelation function. The process of chaotic light optimization model based on MOGA is shown in Fig 6.

The directional preference algorithm is a method used to process directional data in a dataset. The basic idea of this algorithm is to identify the main directions in the dataset through calculation or statistical methods based on the directional information in the data. The direction preference algorithm is commonly used to calculate and analyze the direction vector, angle, or direction distribution in data and determine the main direction or trend of the data. The direction preference algorithm can calculate indicators such as the average value of the direction vector, the standard deviation of the direction vector, and the direction histogram to understand the direction distribution of the data. The direction preference algorithm utilizes the angular relationship between individuals in a population for calculation. The direction preference algorithm can incorporate the preference information of decision-makers into the algorithm, making the population approach the preference region of decision-makers. There are three main methods for guiding population evolution through preference information: interactive, prior, and posterior methods. When calculating, the use of the preference mechanism of angles instead of the crowding ranking algorithm not only speeds up the search speed but also finds the Pareto optimal solution corresponding to the preference point more easily. The search direction of the population is determined by setting preference points, and formula (15) is the search direction formed by the origin and preference points.


$$Angle(\vec{a},\vec{r})=\text{arccos}\left(\frac{\vec{a}\cdot \vec{r}}{\left|\vec{a}\right|\cdot \left|\vec{r}\right|}\right)$$ (15)

**Fig 7:**
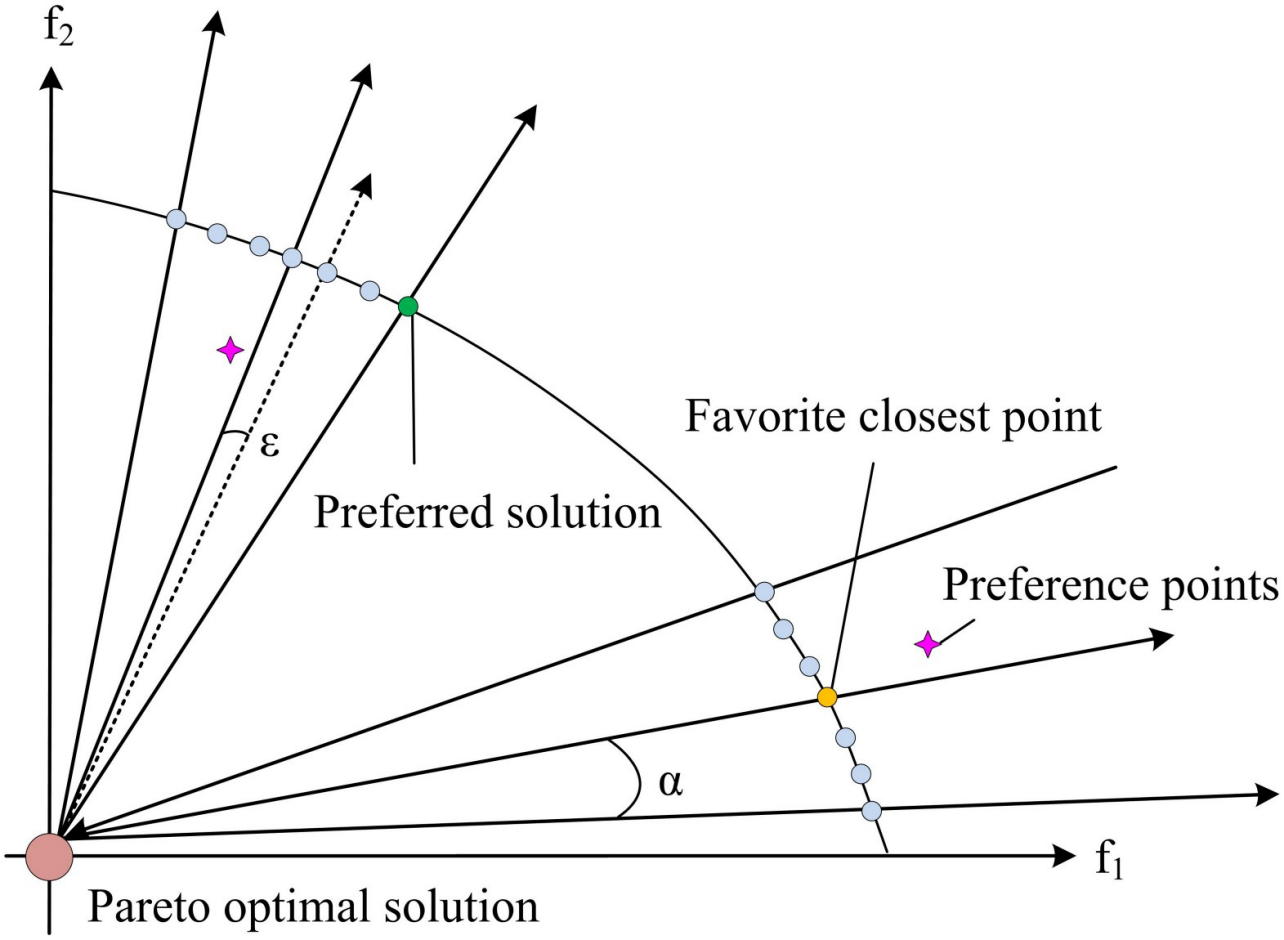
Schematic diagram of angle preference solution for minimizing optimization problems.

In formula (15), $\vec{a}$ represents the origin. $\vec{r}$ represents a preference point. Using the perspective of each individual as the second criterion for forming the Pareto optimal solution in search not only guides the search toward the desired region by combining preference information, but also enhances the strength of selection. In minimization optimization problems, Pareto is usually set as the origin. The search is conducted through the set preference points, lower bound values, and other information in Fig 7.

A small peak of the autocorrelation function indicates good confidentiality of chaotic light. The optimized individual with a peak value of 0.3 has weak suppression of the delay characteristics of chaotic light. But the optimized individual has a large effective bandwidth and maximum Lyapunov exponent, which can be applied to high-speed information transmission in complex regions. The optimized individual with a peak value of 0.2 has good suppression of delay characteristics and a wide range of applicability. The optimized individual with a peak value less than 0.2 is considered to completely suppress the delay characteristics. Therefore, after NSGA-II calculation, the research introduces directional preference algorithm for local optimization to obtain better SLS parameter configuration and find more excellent SLS parameter schemes at peak values of 0.1, 0.2, and 0.3. The optimization solution space for the desired optimization direction ${\vec{F}}_{r}=[f_{1r},f_{2r},f_{3r}]$ is composed of the origin and three representative optimization solution values (*f*_1*r*_, *f*_2*r*_, *f*_3*r*_) selected. The origin is simultaneously connected with other optimized individual *k* to form ${\vec{F}}_{k}=[f_{1k},f_{2k},f_{3k}]$. Formula (16) represents the vector and objective optimization direction.


$$\theta =\text{arccos}\left(\frac{{\vec{F}}_{k}\cdot {\vec{F}}_{r}}{\left|{\vec{F}}_{k}\right|\cdot \left|{\vec{F}}_{r}\right|}\right)$$ (16)

In summary, a MOGA-based chaotic light performance optimization model has been established to optimize the performance of chaotic light. Firstly, the optimization parameters and scope are determined. Then NSGA-II is selected in MOGA to preserve better solutions from being destroyed by genetic mutation operations. And directional preference algorithm is introduced to make the population approach the preference region of the decision-maker. Finally, the peak value of the autocorrelation function is selected as the evaluation indicator.

## 4. Analysis of chaotic light optimization effect based on improved multi-objective genetic algorithm

In this study, an innovative power control algorithm for semiconductor laser-driven system was proposed, and MOGA and the direction preference algorithm were used to optimize the chaotic optical performance. However, the effectiveness of the model needs further verification. The research mainly analyzed from two aspects. Firstly, the effectiveness of the power control algorithm for the semiconductor laser driving system was analyzed. Secondly, the effectiveness of the improved MOGA-based chaotic optical performance optimization model was analyzed.

### 4.1. Effect analysis of control algorithm for semiconductor laser drive system

**Fig 8:**
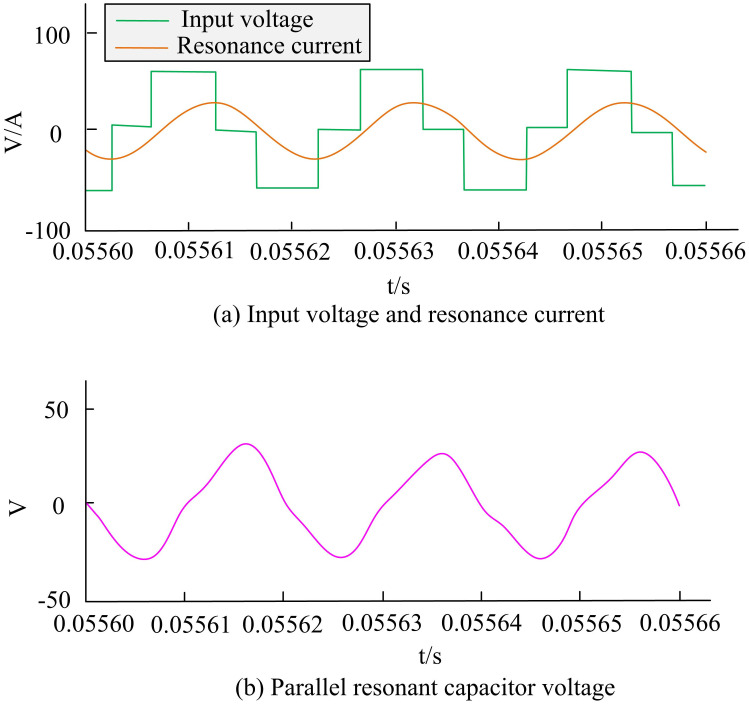
Simulation waveform of LCC resonant converter PID algorithm.

The simulation model of the main power supply LCC resonant converter was built using Matlab/Simulink software, and simulation experiments were conducted. The input current was set to 40A, the input voltage was 60V, and the switching frequency was 51.5kHz. In Fig 8, the PID control strategy output stable voltage and current, and the range of current and voltage fluctuations was small. The maximum voltage of the parallel resonant capacitor reached 36V, but the resonant current was large, with a peak value of 41A. Excessive resonant current easily led to high current and voltage stress in the switching tube, poor controllability of the system, insufficient regulation ability of output current, thus affecting the performance and service life of LCC resonant converters [29].

**Fig 9:**
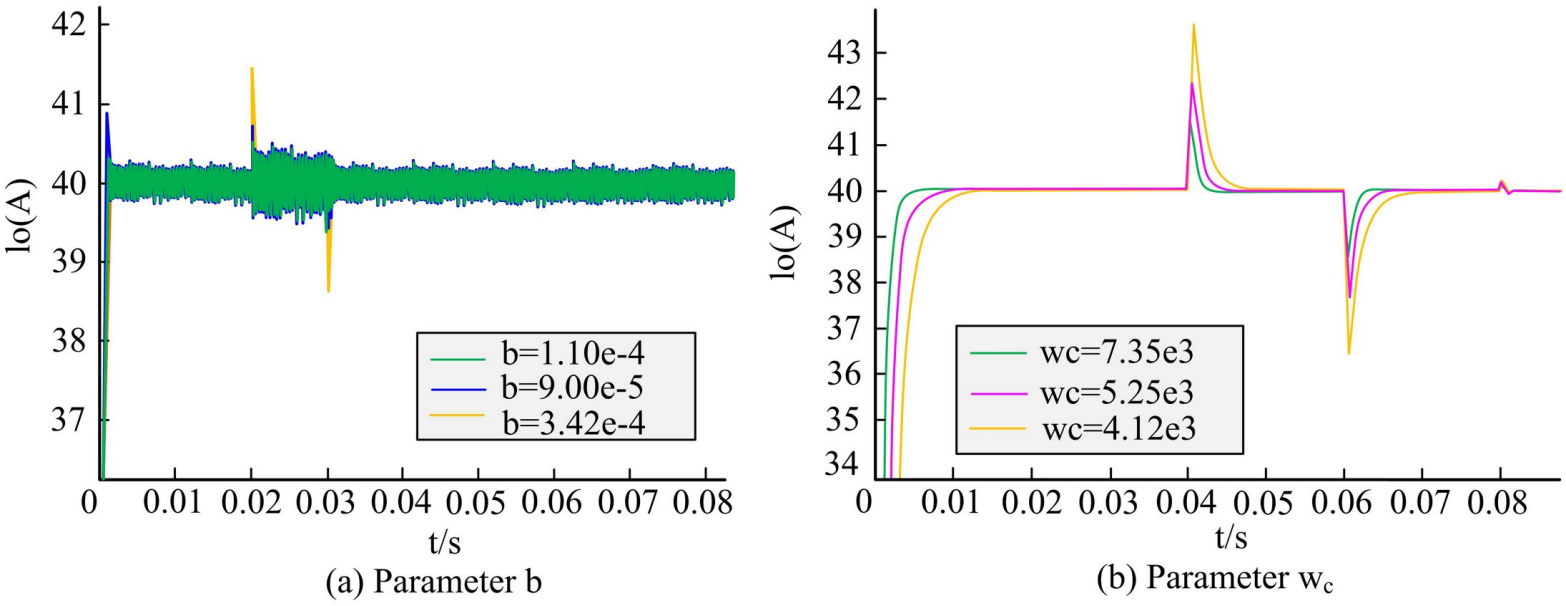
The influence of parameter changes on the output current of LADRC algorithm.

Experiments were conducted in a simulation model of the main power supply LCC resonant converter to verify the influence of parameters *b* and *ω*_*c*_ on the performance of LADRC. Fig 9 shows the effect of two parameters on the output current. From Fig 9(a), under different parameters *b*, the output current of the LCC resonant converter varied. When *b* was 3.42e-4, the impulse current was the highest. These confirmed that *b* had a certain impact on the ripple current and adjustment time of the output current of the LCC resonant converter. A larger *b* resulted in higher impulse current and weaker anti-interference ability. From Fig 9(b), the parameter *ω*_*c*_ had a certain impact on the response speed of the output current of the LCC resonant converter. A larger *ω*_*c*_ resulted in faster response speed, but the impact current was greater when disturbed, resulting in poor system stability.

**Fig 10:**
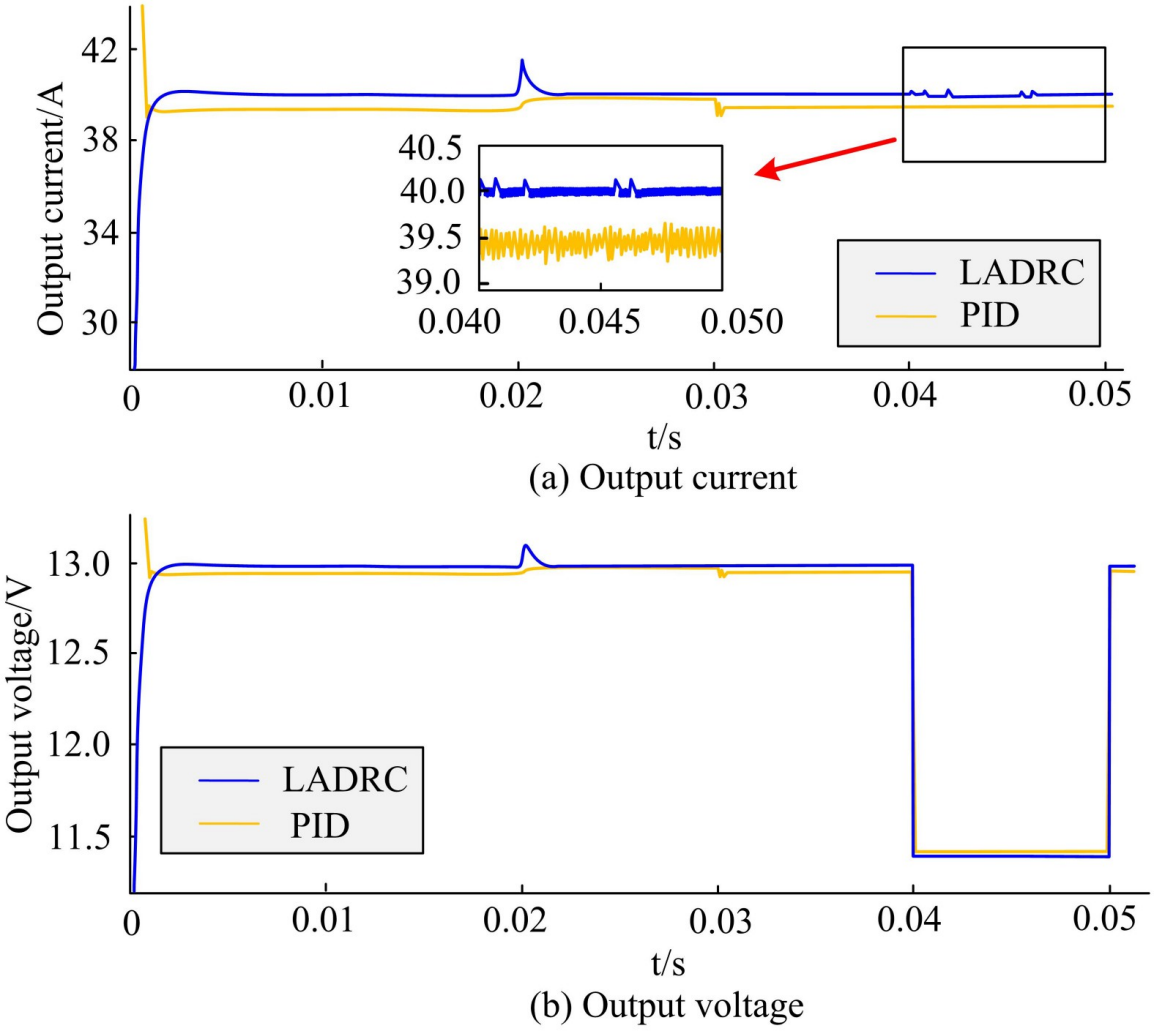
Comparison results of output current and voltage between LADRC algorithm and PID algorithm.

LADRC was compared with PID to verify the superiority of the proposed power control algorithm for the semiconductor laser driving system. The input voltage was set to 60V, the input current was 40A, and the switching frequency was 51.5kHz. In the range of 0.02s to 0.03s, the input voltage was suddenly changed to 80V. In the range of 0.04s to 0.05s, the load was adjusted to 60%. Fig 10 shows the comparison results of output current and voltage between these two algorithms. When the input voltage underwent a sudden change, PID had a large impulse current and a fast response speed, with a current ripple coefficient of 0.55%. Although the output current ripple coefficient of the LADRC algorithm increased to 0.42%, the voltage and current maintained at the set values. When the load underwent a sudden change, the stability of the LADRC algorithm was better, the output current was more stable, which had certain feasibility and superiority.

### 4.2. Effect analysis of chaotic optical performance optimization model

**Fig 11:**
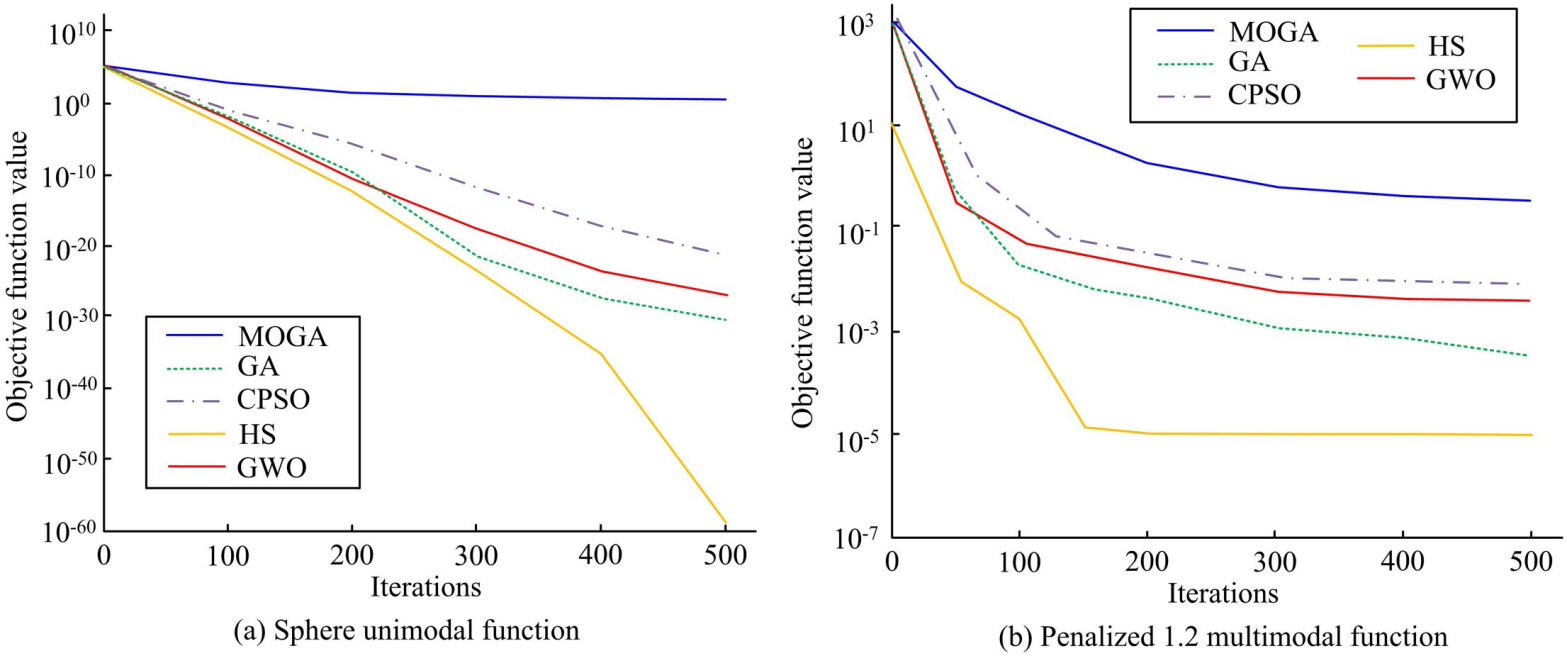
The iteration curve of the test function.

Experiments were conducted using the Sphere unimodal function and Penalized 1.2 multimodal function, with a population size of 30 and an iteration count of 500 to verify the performance of the proposed MOGA. The proposed MOGA was compared with traditional GA, Constrained Particle Swarm Optimization (CPSO), Harmony Search (HS), and Grey Wolf Optimization (GWO) algorithms. The results are shown in Fig 11. From Fig 11(a), among the five algorithms, the proposed MOGA algorithm showed significant advantages in convergence speed and accuracy, tending to converge after 200 iterations, while the HS algorithm performed the worst. From Fig 11(b), the MOGA algorithm had good performance in multimodal functions and tended to converge at 300 iterations. The results showed that the proposed MOGA algorithm not only had better convergence speed than the other four algorithms, but also could find better solutions, which had certain feasibility and superiority.

**Fig 12:**
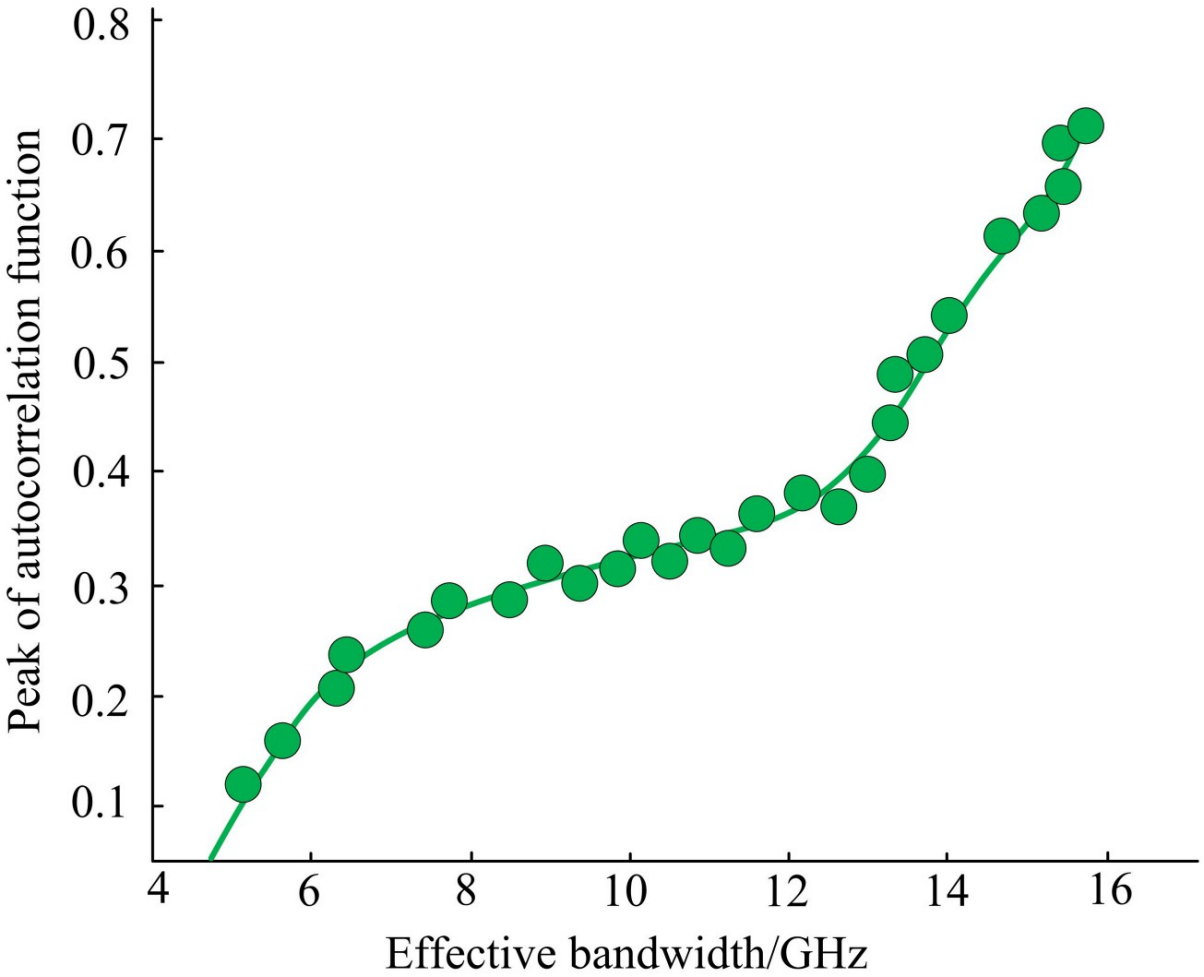
The functional relationship between autocorrelation and bandwidth in the 1000th generation optimized population.

The optimization parameters were taken as initial values within the optimization range by optimizing the combination of SLS parameters to output high-performance chaotic light. All parameters were substituted into the algorithm. Fig 12 shows the functional relationship between autocorrelation and bandwidth in the 1000th generation optimized population. From Fig 12, there was no significant relationship between the effective bandwidth and the maximum Lyapunov exponent. The peak of the autocorrelation function was positively correlated with the maximum Lyapunov exponent. In addition, there was a mutual constraint relationship between the effective bandwidth and the peak autocorrelation function. Therefore, if the bandwidth increased, the suppression of delay characteristics inevitably decreased.

**Fig 13:**
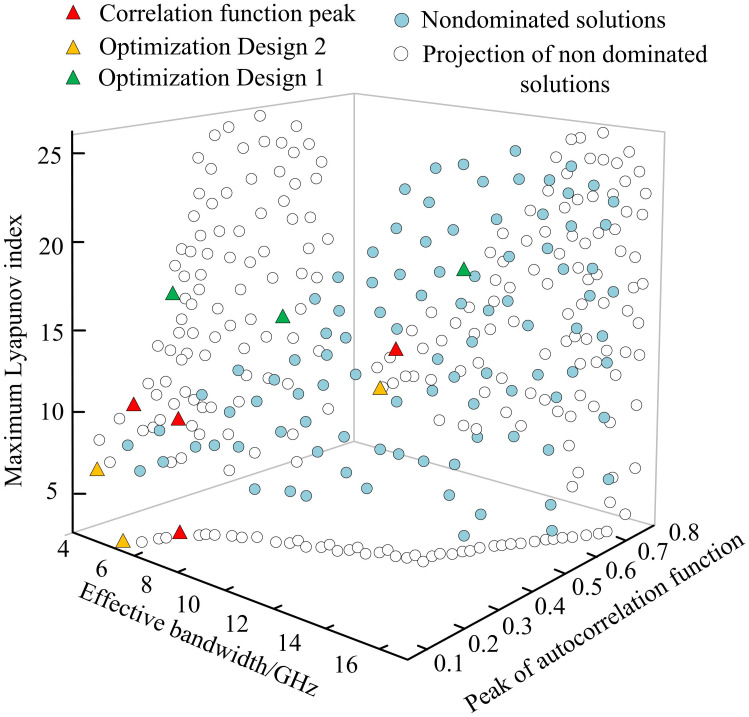
Optimization individuals selected for the 1000th generation optimization population.

Fig 13 shows the optimized individuals selected from the 1000th generation optimized population. Optimization design 1 indicates a peak autocorrelation function of 0.1. Optimization design 2 indicates a peak autocorrelation function of 0.2. The maximum Lyapunov exponent was positively correlated with the peak of the autocorrelation function, while the effective bandwidth and the maximum Lyapunov exponent had no significant relationship. There was a clear mutual constraint between the effective bandwidth and the peak autocorrelation function, so increasing bandwidth inevitably led to a decrease in the suppression of delay characteristics. In addition, when the peak of the autocorrelation function was less than 0.2, the function between bandwidth and autocorrelation was relatively steep. The results indicated that there was no significant functional relationship between the two optimization objectives in the entire state. However, the maximum Lyapunov exponent in the Pareto surface was positively correlated with the peak autocorrelation function in the range of 0–0.4 peak autocorrelation function, which had no significant relationship with the effective bandwidth.

**Fig 14:**
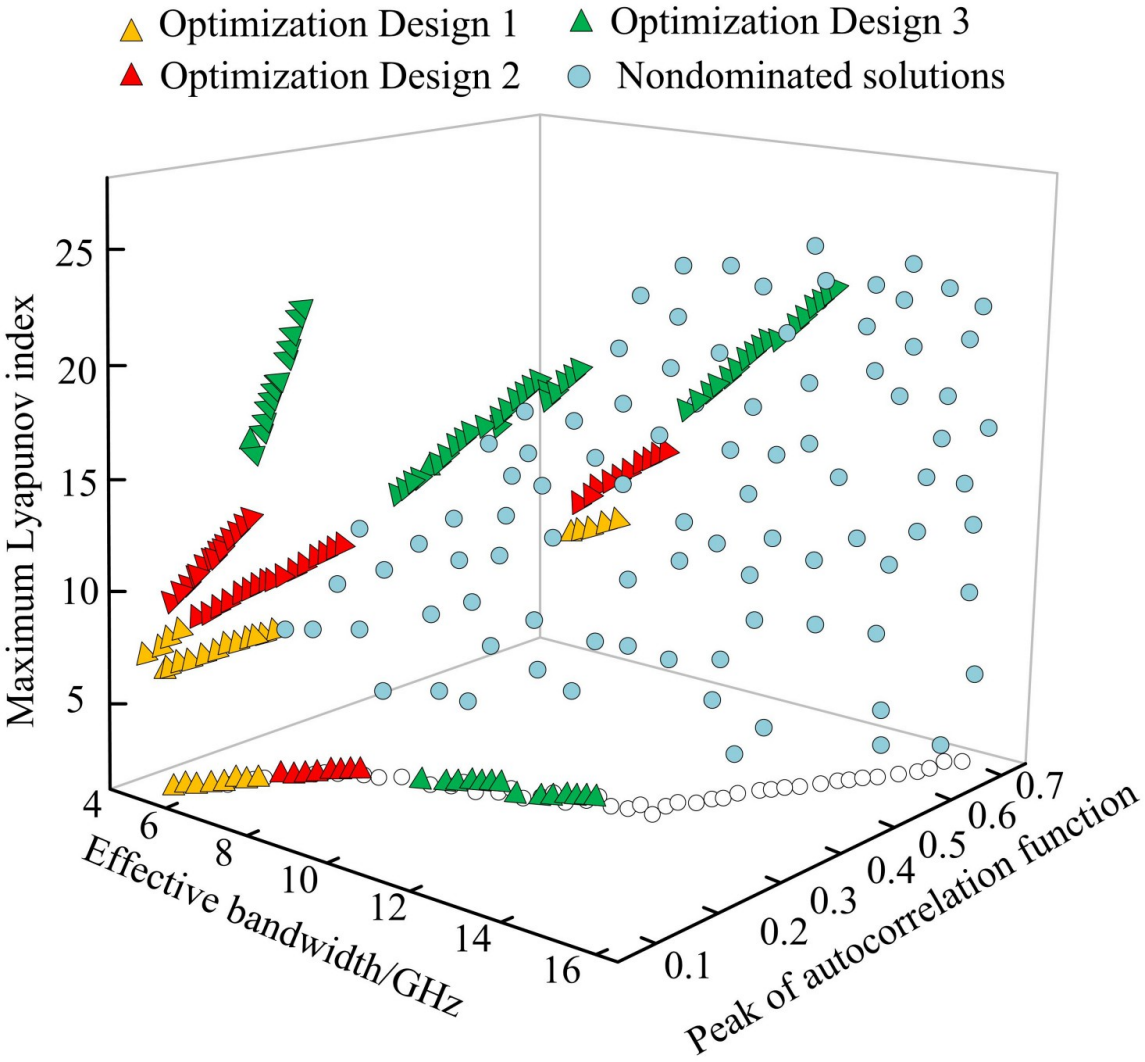
Population optimized locally through angle preference algorithm.

In Fig 14, the direction preference algorithm was used to search for optimization solutions within the target region, i.e. the optimization solutions at the peak autocorrelation functions of 0.1, 0.2, and 0.3. All individuals gathered in the non-dominant frontier, and the peak autocorrelation function and the effective bandwidth were conflicting. By comparison, the direction preference algorithm further provided more valuable solutions on the basis of NSGA-II, and this algorithm did not exceed the nondominated frontier obtained by NSGA-II, which had certain feasibility and effectiveness.

The SLS parameter configuration was reselected for simulation inversion after model optimization to verify the effectiveness of the improved MOGA-based chaotic optical performance optimization model. Table 2 shows the selected parameter configurations.

**Table 2:** Parameter configuration re selected after optimization.

Parameter	Peak autocorrelation function	
	0.1	0.2
Feedback intensity/ns	41.2	8.9
Injected current	1.2	1.27
Detuning frequency/GHz	-17.94	-1.67
Linewidth enhancement factor	4.9	4.99
Laser external cavity length/m	0.1	0.103

**Fig 15:**
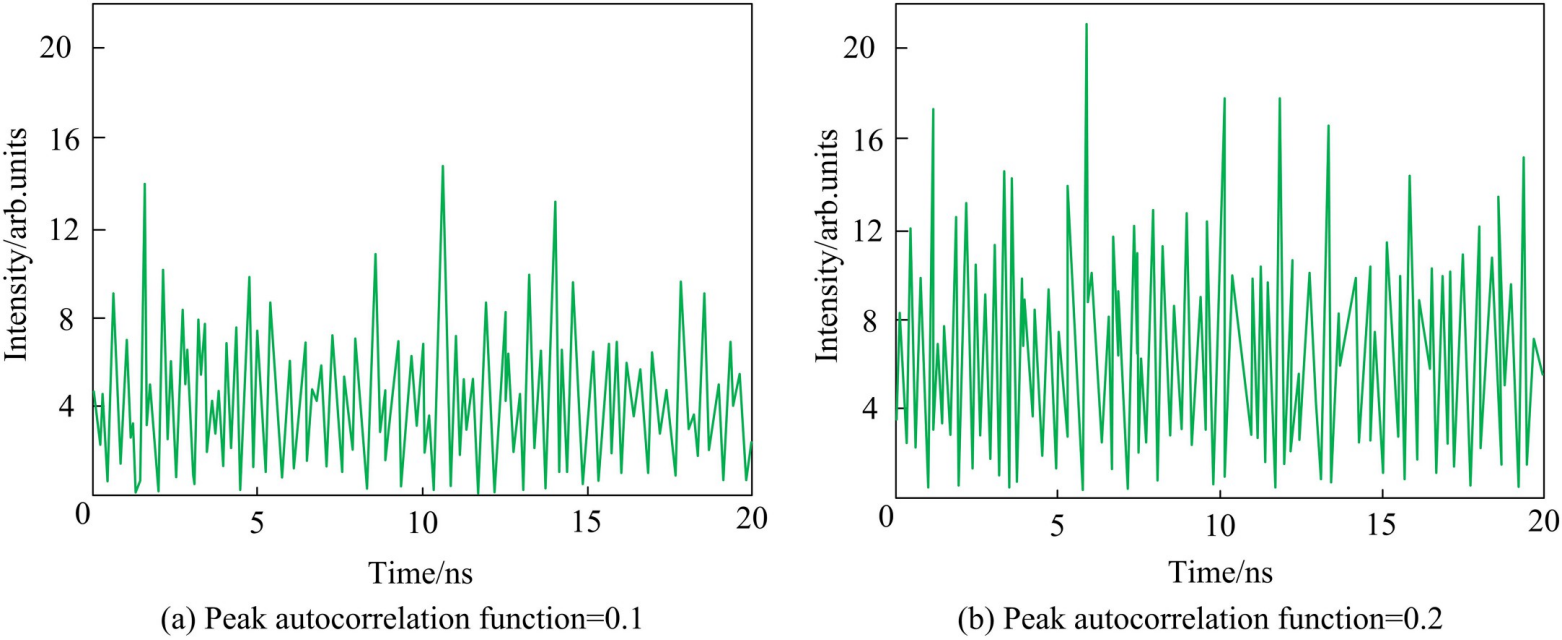
Time-series of two design optimization schemes.

The results obtained by the proposed model were validated through simulation inversion using the parameter configuration of SLS. Fig 15 shows the time waveform of the response to a semiconductor laser under two types of autocorrelation function peaks. When having a large maximum Lyapunov exponent, the chaotic light provided a more complex time series. When the peak value of the autocorrelation function was equal to 0.1, the delay characteristic was well suppressed, and the delay characteristic almost disappeared. Through simulation inversion, the peak autocorrelation function and bandwidth in the SLS optimization scheme were suppressing mutually, proving the effectiveness and feasibility of the improved MOGA-based chaotic optical performance optimization model. In addition, when the peak value of the correlation function was equal to 0.2, the delay characteristics of chaotic light were well suppressed and had strong signal bandwidth and complexity, being regarded as the global optimal solution.

The feasibility of the proposed chaotic optical performance optimization model was verified in this study. However, due to limited experimental level and conditions, the design of an experimental prototype cannot fully represent all the characteristics of the high-power semiconductor laser drive system, and the number of experiments is relatively small, which may lead to some randomness.

## 5. Conclusion

With the development of technology, communication methods become increasingly diverse, and communication security also receives increasing attention. A power control algorithm for semiconductor laser drive system was proposed to effectively optimize the chaotic optical performance of SLS output, and a chaos optical performance optimization model based on MOGA was established. These results indicated that the response speed of PID was faster, but the current and voltage stress of the switch tube were increased. The parameter *b* affected the output current of the LCC resonant converter, and a larger *b* resulted in weaker anti-interference ability. The parameter *ω*_*c*_ had a certain impact on the response speed of the output current of the LCC resonant converter. The larger *ω*_*c*_ resulted in faster response speed, but the impact current was greater when disturbed, resulting in poorer system stability. When the input voltage underwent a sudden change, the current ripple coefficient of the PID was 0.55%, while LADRC ensured that the voltage and current were maintained at the set values. When the load underwent a sudden change, the output current of LADRC became more stable. The direction preference algorithm further provided more valuable solutions on the basis of NSGA-II and did not exceed the nondominated frontier obtained by NSGA-II. When the peak value of the autocorrelation function was equal to 0.1, the delay characteristic was well suppressed, and the delay characteristic almost disappeared. When the peak value of the autocorrelation function was equal to 0.2, the delay characteristics of chaotic light was effectively suppressed with strong signal bandwidth and complexity, being regarded as the global optimal solution. In summary, the model constructed by the research has certain feasibility and effectiveness. The proposed LADRC algorithm has good stability and more stable output current. The MOGA algorithm not only has better convergence speed than the other four algorithms, but also finds better solutions at a higher level. Therefore, the SLS optimized by the model has better stability and performance in chaotic light output compared with the current technological level, which can be better applied in high-speed data transmission. The innovation of this study lies in proposing a power control algorithm for semiconductor laser drive system to improve the stability, spectrum width, and dynamic range of chaotic light. The noise and nonlinear effects can be reduced. Meanwhile, multi-objective genetic optimization algorithm is used to preserve better solutions, and the direction preference algorithm is used to make the population approach the preference area of decision-makers. However, the SLS used in this study is relatively single, which may affect the actual application effect of the model. Therefore, in the following research, further MOP should be conducted on other SLS to further verify the adaptability and effectiveness of the proposed method and better improve the chaotic optical performance of the SLS output.

## Supporting information

S1 File (DOCX)

## Abbreviations

GA: Genetic Algorithm
LADRC: Linear Active Disturbance Rejection Control
MOGA: Multi-objective Genetic Algorithm
MOP: Multi-objective Optimization
NSGA-II: Nondominated Sorting Genetic Algorithm-II
SLS: Semiconductor Laser System

## References

[1] Dong D. and Feng H., "Design and use of a wireless temperature measurement network system integrating artificial intelligence and blockchain in electrical power engineering," PLoS One, vol. 19, no. 1, pp. e0296398, Jan. 2024, doi: 10.1371/journal.pone.0296398 38165871 PMC10760662

[2] Zhang L. and Liu Z., "Research on technology prospect risk of high-tech projects based on patent analysis," PLoS One, vol, 15, no. 10, pp. e0240050, Oct. 2020, doi: 10.1371/journal.pone.0240050 33017432 PMC7535056

[3] Güven A. F. and Samy M. M., "Performance analysis of autonomous green energy system based on multi and hybrid metaheuristic optimization approaches," ENERG CONVERS MANAGE, no. 269, pp. 116058, Oct. 2022, doi: 10.1016/j.enconman.2022.116058

[4] Barakat S., Emam A. and Samy M. M., "Investigating grid-connected green power systems’ energy storage solutions in the event of frequent blackouts," ENERGY REP, no. 8, pp. 5177–5191, Nov. 2022, doi: 10.1016/j.egyr.2022.03.201

[5] Suginoma H., Owada R., Katano-Toki A., Mori A., Fujioka J. and Nakamura K., "Non-fibril form but not fibril form of human islet amyloid polypeptide 8–20 changes brain functions in mice," PLoS One, vol. 19, no. 1, pp. e0296750, Jan. 2024, doi: 10.1371/journal.pone.0296750 38181010 PMC10769099

[6] Lu C., Huang Y., Meng L, Gao L., Zhang B., and Zhou J., "A Pareto-based collaborative multi-objective optimization algorithm for energy-efficient scheduling of distributed permutation flow-shop with limited buffers," Robot Cim-Int. Manuf., vol. 74, no. 2, pp. 102277–102279, Apr. 2022, doi: 10.1016/j.rcim.2021.102277

[7] Mokhtara C., Negrou B., Settou N., Settou B. and Samy M. M., "Design optimization of off-grid Hybrid Renewable Energy Systems considering the effects of building energy performance and climate change: Case study of Algeria," Energy, vol. 219, pp. 119605, Mar. 2021, doi: 10.1016/j.energy.2020.119605

[8] Tian Y., Si L., Zhang X., Cheng R., He C., Tan K. C., et al., "Evolutionary large-scale multi-objective optimization: A survey," Acm. Comput. Surv., vol. 54, no. 8, pp. 1–34, Oct. 2021, doi: 10.1145/3470971

[9] Wang J., Jia G., Lin J., and Hou Z., "Cooperative task allocation for heterogeneous multi-UAV using multi-objective optimization algorithm," J. Cent. South Univ., vol. 27, no. 2, pp. 432–448, Feb. 2020, doi: 10.1007/s11771-020-4307-0

[10] Martinez-Rico J., Zulueta E., de Argandoña I. R., Fernandez-Gamiz U., and Armendia M., "Multi-objective optimization of production scheduling using particle swarm optimization algorithm for hybrid renewable power plants with battery energy storage system," J. Mod. Power Syst. Clean Energy, vol. 9, no. 2, pp. 285–294, Mar. 2020, doi: 10.35833/MPCE.2019.000021

[11] Dinh-Cong D., and Nguyen-Thoi T., "An effective damage identification procedure using model updating technique and multi-objective optimization algorithm for structures made of functionally graded materials," Eng. Comput-Germany, vol. 39, no. 2, pp. 1229–1247, Apr. 2023, doi: 10.1007/s00366-021-01511-7

[12] Jangir P., Buch H., Mirjalili S., and Manoharan P., "MOMPA: multi-objective marine predator algorithm for solving multi-objective optimization problems," Evol. Intell., vol. 16, no. 1, pp. 169–195, Feb. 2023, doi: 10.1007/s12065-021-00649-z

[13] Tazay A. F., Samy M.M., and Barakat S. A, "Techno-Economic Feasibility Analysis of an Autonomous Hybrid Renewable Energy Sources for University Building at Saudi Arabia," J. Electr. Eng. Technol., vol. 15, pp. 2519–2527, Sep.2020, doi: 10.1007/s42835-020-00539-x

[14] Zhang M., and Wang Y., "Review on chaotic lasers and measurement applications," J. Lightwave Technol., vol. 39, no. 12, pp. 3711–3723, Dec. 2020, doi: 10.1109/JLT.2020.3043829

[15] Gao Z., Ma Z., Wu S., Gao H., Wang A., Fu S., et al., "Physical secure key distribution based on chaotic self-carrier phase modulation and time-delayed shift keying of synchronized optical chaos," Opt. Express, vol. 30, no. 13, pp. 23953–23966, Jun. 2022, doi: 10.1364/OE.460773 36225066

[16] Annovazzi-Lodi V., Donati S., and Scire A., "Synchronization of chaotic injected-laser systems and its application to optical cryptography," IEEE J. Quantum. Elect., vol. 32, no. 6, pp. 953–959, Jun. 1996, doi: 10.1109/3.502371

[17] Roes M. G. L., Duarte J. L., and Hendrix M. A. M., "Disturbance observer-based control of a dual-output LLC converter for solid-state lighting applications," IEEE T. Power. Electr., vol. 26, no. 7, pp. 2018–2027, Jul. 2011, doi: 10.1109/TPEL.2010.2101086

[18] Buccella C., Cecati C., Latafat H., Pepe P., and Razi K., "Observer-based control of LLC DC/DC resonant converter using extended describing functions," IEEE T. Power. Electr., vol. 30, no. 10, pp. 5881–5891, Oct. 2015, doi: 10.1109/TPEL.2014.2371137

[19] Suresh K. and Parimalasundar E., "A Modified Multi Level Inverter With Inverted SPWM Control," IEEE CAN J ELECT COMPUT E, vol. 45, no. 2, pp. 99–104, Apr. 2022, doi: 10.1109/ICJECE.2022.3150367

[20] Falehi A. D. and Torkaman H., "Robust fractional-order super-twisting sliding mode control to accurately regulate lithium-battery/super-capacitor hybrid energy storage system," INT J ENERG RES, vol. 45, no. 13, pp. 18590–18612, Jul. 2021, doi: 10.1002/er.7045

[21] Falehi A. D. and Torkaman H., "Promoted supercapacitor control scheme based on robust fractional-order super-twisting sliding mode control for dynamic voltage restorer to enhance FRT and PQ capabilities of DFIG-based wind turbine. J Energy Storage, vol. 42, pp.102983, Oct. 2021, doi: 10.1016/j.est.2021.102983

[22] Falehi A. D., "Half-cascaded multilevel inverter coupled to photovoltaic power source for AC-voltage synthesizer of dynamic voltage restorer to enhance voltage quality.": INT J NUMER MODEL EL, vol. 34, no. 5, pp. e2883, Mar. 2021, doi: 10.1002/jnm.2883

[23] Zhao J., Chen Y., Zeng J. and Liu J., "A Hybrid Nine-Level Inverter With Reduced Components and Simplified Control," IEEE J EM SEL TOP P, vol. 10, no. 4, pp. 4498–4508, Aug. 2022, doi: 10.1109/JESTPE.2022.3152994

[24] Lu M., Dhople S. and Johnson B., "Benchmarking Nonlinear Oscillators for Grid-Forming Inverter Control," IEEE T POWER ELECTR, vol. 37, no. 9, pp. 10250–10266, Sept. 2022, doi: 10.1109/TPEL.2022.3162530

[25] Choi H., Phoulady A., Hoveida P., May N., Shahbazmohamadi S. and Tavousi P., "Automated, real-time material detection during ultrashort pulsed laser machining using laser-induced breakdown spectroscopy, for process tuning, end-pointing, and segmentation," PLoS One, vol. 19, no. 1, pp. e0290761, Jan. 2024, doi: 10.1371/journal.pone.0290761 38215075 PMC10786384

[26] Abdelfattah H., Esmail M., Kotb S. A., Mahmoud M. M., Hussein H. S., Wapet D. E. M., et al., "Optimal controller design for reactor core power stabilization in a pressurized water reactor: Applications of gold rush algorithm," PLoS One, vol. 19, no. 1, pp. e0296987, Jan. 2024, doi: 10.1371/journal.pone.0296987 38277423 PMC10817221

[27] Zhang Q., Hu J., Liu Z., and Duan J., "Multi-objective optimization of dual resource integrated scheduling problem of production equipment and RGVs considering conflict-free routing," PLoS One, vol. 19, no. 1, pp. e0297139, Jan. 2024, doi: 10.1371/journal.pone.0297139 38277415 PMC10834064

[28] Lin L., Wang Z., Tian L., Wu J. and Wu W., "A PSO-based energy-efficient data collection optimization algorithm for UAV mission planning," PLoS One, vol. 19, no. 1, pp. e0297066, Jan. 2024, doi: 10.1371/journal.pone.0297066 38241422 PMC10798525

[29] Lyu Z., Wu L., Yi J. and Yang S., "Hybrid Frame-Based Current Control Scheme for LC-Equipped PMSM With Non-Sinusoidal Back-EMF," IEEE T POWER ELECTR, vol. 38, no. 5, pp. 5994–6004, May. 2023, doi: 10.1109/TPEL.2023.3241625

