# Supplementary material for: Optimization of chaotic light output in semiconductor laser systems based on multi-objective optimization algorithm
Source: PLoS One. 2024 Apr 11;19(4):e0301630. doi: 10.1371/journal.pone.0301630 (PMC11008891; doi:10.1371/journal.pone.0301630)
Supplement: S1 File — (DOCX) [file pone.0301630.s001.docx]

**The Data in Figure 8**

| Time/s | 0.05561 | 0.05562 | 0.05563 | 0.05564 | 0.05565 | 0.05566 |
| --- | --- | --- | --- | --- | --- | --- |
| Input voltage | 60V | -60V | 60V | -60V | 60V | -60V |
| Resonance current | 20A | -20A | 20A | -20A | 20A | -20A |
| Parallel resonant capacitor voltage | 0V | 0V | 0V | 0V | 0V | 0V |

**The Data in Figure9**

| Time/s | 0.01 | 0.02 | 0.03 | 0.04 | 0.05 | 0.06 | 0.07 | 0.08 |
| --- | --- | --- | --- | --- | --- | --- | --- | --- |
| b=1.10e-4 | 39.8A | 40.0A | 40.6A | 40.1A | 39.9A | 40.1A | 40.0A | 39.8A |
| b=9.00e-5 | 40.1A | 40.8A | 39.4A | 40.0A | 40.2A | 40.0A | 39.8A | 39.9A |
| b=3.42e-4 | 40.1A | 41.3A | 38.7A | 40.0A | 40.1A | 40.0A | 39.8A | 39.9A |
| w_c_=7.35e3 | 40.0A | 40.0A | 40.0A | 41.8A | 40.0A | 38.8A | 40.0A | 40.1A |
| w_c_=5.25e3 | 40.0A | 40.0A | 40.0A | 42.6A | 40.0A | 37.6A | 40.0A | 40.1A |
| w_c_=4.12e3 | 39.8A | 40.0A | 40.0A | 43.5A | 40.0A | 36.5A | 40.0A | 40.1A |

**The Data in Figure10**

| - | Time/s | 0.01 | 0.02 | 0.03 | 0.04 | 0.05 |
| --- | --- | --- | --- | --- | --- | --- |
| Output current/A | LADRC | 40.0A | 42.0A | 38.9A | 40.2A | 40.0A |
|  | PID | 38.6A | 38.5A | 38.5A | 39.6A | 39.3A |
| Output voltage/V | LADRC | 13.0V | 13.2V | 13.0V | 11.3V | 11.3V |
|  | PID | 12.8V | 12.9V | 12.8V | 11.4V | 11.4V |

**The Data in Figure11**

| - | Iteration | 100 | 200 | 300 | 400 | 500 |
| --- | --- | --- | --- | --- | --- | --- |
| Sphere unimodal function | GA | 10^-2^ | 10^-9^ | 10^-22^ | 10^-27^ | 10^-30^ |
|  | CPSO | 10^-1^ | 10^-5^ | 10^-13^ | 10^-17^ | 10^-21^ |
|  | HS | 10^-3^ | 10^-13^ | 10^-23^ | 10^-34^ | 10^-59^ |
|  | GWO | 10^-2.2^ | 10^-10^ | 10^-8^ | 10^-24^ | 10^-28^ |
|  | MOGA | 10^2^ | 10^1^ | 10^1^ | 10^1^ | 10^0^ |
| Penalized 1.2 multimodal function | GA | 10^-1.5^ | 10^-2.1^ | 10^-2.6^ | 10^-2.8^ | 10^-3^ |
|  | CPSO | 10^-0.3^ | 10^-1.3^ | 10^-1.5^ | 10^-1.5^ | 10^-1.5^ |
|  | HS | 10^-2.5^ | 10^-4.8^ | 10^-4.8^ | 10^-4.8^ | 10^-4.8^ |
|  | GWO | 10^-1.3^ | 10^-1.7^ | 10^-2^ | 10^-2.1^ | 10^-2.1^ |
|  | MOGA | 10^1.7^ | 10^0.4^ | 10^0.2^ | 10^0.1^ | 10^0.1^ |

**The Data in Figure12**

| Effective bandwidth/GHz | 6 | 8 | 10 | 12 | 14 | 16 |
| --- | --- | --- | --- | --- | --- | --- |
| Peak of autocorrelation function | 0.20 | 0.08 | 0.33 | 0.37 | 0.56 | 0.74 |

**The Data in Figure15**

| Time/ns | 0 | 5 | 10 | 15 | 20 |
| --- | --- | --- | --- | --- | --- |
| Peak of autocorrelation function=0.1 | 4.4 | 7.2 | 2.8 | 5.1 | 2.4 |
| Peak of autocorrelation function=0.2 | 3.1 | 1.9 | 17.8 | 1.8 | 5.7 |
